# Editorial

**Published:** 1846-11

**Authors:** 


### THE MEDICAL EXAMINER.

### PHILADELPHIA, NOVEMBER, 1846.

### PHILADELPHIA MEDICAL COLLEGES.

The period for the commencement of the courses of instruction in our Medical schools is near at hand, and physicians and students are hourly coming into the city to join the classes; how large these will be, it is yet impossible to tell, but from all the indications before us, we shall not be surprised if the numbers should exceed those of last winter. The season thus far is mild, and the city healthy, and every thing bodes a pleasant and profitable winter for our guests.

*The Annalist; a Record of Practical Medicine, in the city of New York. Edited by* WILLIAM C. ROBERTS, M. D.. etc.

This is a new Medical Journal, of which we have received the first two numbers. It is to be published in New York, on the first and fifteenth of every month, and contain twenty-four pages of matter.

The editor, in his inaugural address, remarks: "On presenting to the profession the first number of a new medical periodical, the editor naturally feels that the untimely end of its many predecessors will be remembered, and that a similar speedy decline will be predicted of this." He adds "his own assurances to those of his publisher," however, "that such arrangements have been made as place its continuance beyond the contingency of failure"—to which we heartily respond *amen.* The editor declares "His aim is to make the Annalist an arena in which all may meet on equal terms, to confer upon the great subject which occupies their every thought, and forms the business of their lives. All who adorn the medical name by purity of private and professional character, and pursue steadily the straight, but rough and narrow path of Legitimate Medicine, shall be welcome to the use of its pages; but to no others will they be open."

These sentiments manifest an honest purpose, and must secure for our brother the best wishes of all the true friends of our noble profession.

---

NEW YORK MEDICAL SOCIETY AND QUACKERY.

"*Shall the Charter of the New York Medical Society be surrendered?*"

In a late number of the New York Medical and Surgical Reporter, (Oct. 10th, 1846,) this question is discussed with much point and spirit by Dr. Calkins, as a means of getting rid of all connection and fraternization with certain members, who it appears are degrading the profession by their empirical conduct. As a legally constituted body, it cannot expurgate itself by the removal of unworthy members, without the interposition of the judiciary, as we infer from the writer's remarks—the power to exclude not being co-ordinate with that to elect. This necessarily is attended with many difficulties, and legislative action would be required to render the procedure practically useful. The writer appears to think it would not be expedient to deal with Homœopathists and such as pursue a course more or less popular.

"The crude and wayward convictions of the multitude, preposterous as they would appear to the professional eye, must be treated as we would handle the currency, estimating its denominational as well as its intrinsic value. In a science of all the most recondite, and amidst theories the most discordant and latitudinarian, how in reason can the popular mind be expected of itself to discriminate, and choose the expedient and safe, to the rejection of the doubtful or the hazardous?

"If, indeed, a flagrant breach of morality comes under our cognizance, if a course of practice is pursued palpably clashing with the primary laws of rectitude and honor, is one chargeable with peculation or other fraud, is the reckless vender of damnable nostrums seen fleeing for shelter to the broad seal of our Society, let us bring him up for sentence, and post him as a renegade and an outlaw. Who shall gainsay the right, or be captiously clamorous against its enforcement?"

Homœopathy, of which the writer appears but little enamoured, ought, he thinks, to be put to the test, as in Paris. "We cannot refer to developements made three thousand miles off, but we might institute, as was done in Paris, a mixed commission, with authority and facilities to make a grand comparative experiment and then publish the results. Such is the trial that the homœopathists themselves profess to covet; the very ordeal, I am bold to aver, they of all things would deprecate and shrink from."

If our brethren in New York should undertake to disprove every ridiculous and pernicious scheme that quacks may devise for fleecing the brainless multitude, they will have little time left for science and their patients. What did the mixed commission in Paris accomplish? It proved to the conviction of every disinterested and right thinking mind that homœopathy, practically carried out, is a humbug; and that was just what physicians and intelligent people thought of it before: but did it stop the mouths of the knaves who profited by it, or convince silly ladies and crotchety old gentlemen—people who are governed by faith, and not by reason—a faith which is truly to them "*the evidence of things not seen*"? Not at all. Such people are never convinced, although they are often converted—but it is to some new folly, frequently greater than the first.

The same Journal from which we have derived the above, tells the following queer story. We think such an *accident* could hardly befal either the *Philadelphia Medical Society*, or a Philadelphia College.

"A Way to get a Diploma.—There is a certain advertising quack in this city, who is said to have obtained a diploma from a Medical College, in the following manner. When he first came to this country he commenced peddling about the streets with a small basket on his arm, which barely enabled him to support his family; but meeting with one of his countrymen, an old German physician, who had been well educated, and had enjoyed a good practice, until he became dissipated, which brought upon him disgrace and degradation, and prepared him for baser deeds and meaner assoiations; said physician, on coming to this country and becoming acquainted with our hero of the toy-basket, and his peculiar mind, advised him to become a doctor. How can I do that when I have no previous education? asked the pedlar. The German doctor replied, You must feign that you cannot speak English, and I will interpret for you, but we must not let the Professor know that I am a physician. All preliminary arrangements being made, and the story having been given out, that the Dutch pedlar had been a surgeon in Napoleon's army, ect., he was examined, and his friend interpreted to suit the case.

The result was, *that he passed a pretty fair examination considering his having been educated in another country, and being wholly unacquainted with our language and mode of examination.*

The diploma being obtained, served him as a passport into the Medical Society of the city and county of New York.

We next find his flaring advertisements in nearly all of our city papers, telling of his *celebrated elixirs and wonderful cures.*

This is but one of the impostors, who have crept into this once highly honorable society, composed of the medical profession of this city, but now, alas! how changed.

Can it be wondered at, that those gentlemen who feel a just indignation at seeing themselves and the profession so trampled upon, should now take some active measures to rid themselves of such mountebanks, or dissolve the connection by giving up the charter?

At the next meeting of the Society, we shall expect to see some action brought forward on the subject."

---

*Annual Announcement of the Medical Department of the University of New York.*—Session of 1846–7.

We have rarely met with a circular from a medical school so little in accordance with our notions of good taste and good feeling as this, nor have we seen one that drew so largely upon the credulity of its readers. Not to go too much into particulars, we shall refer to only two or three examples.

The title page is ornamented with a neat wood-engraving of a very fine building appropriated to the *Academical* department of the University, which, beside being a long way from that in which the Medical Lectures are given, is as unlike it as it is unlike any other building in the city—as unlike it as *a granite fronted building with the lower story filled with shops*, is unlike the handsome marble building figured in this publication.

The language employed throughout this Announcement, it seems to us, is eminently hyperbolical. One object appears to run through all its pages, and in every paragraph—that of impressing the reader with the belief that the Medical Department of the University of New York is *the great school cf the country!* Nay, the schools of Europe, as well as those of America, are brought into invidious comparison. In this, however, "the Faculty would not arrogate to themselves superior talents or learning"! What then? "A few years since, and New York was known only as the *Commercial Emporium;* her fame in Medicine had not travelled beyond the confines of her own state; and her Medical classes were insignificant in numbers, composed mostly of students residing in the vicinity of the city." What then has made the wonderful change so complacently dwelt upon, if not the "superior talents and learning" of the Faculty by whom this Announcement is issued? The city was there—the people, the hospitals, the dispensaries, a well organized medical school—every thing except the "Medical Department of the University of New-York"!

The ridiculous fustian about "*building up a national school worthy of the country and the age*," is truly laughable. Why should a school in the city of New York be *national* any more than in Philadelphia, Cincinnati, New Orleans, Boston or Baltimore, and why the school in the Stuyvesant Institute in Broadway, any more than that in Crosby street? Such extravagant pretensions, bombast and self-laudation, are little calculated to gain the esteem and secure the confidence of the thinking public, and least of all, the medical public.

The Faculty in this Announcement boast of a "surplus of the *materiel*" for practical anatomy, and at the same time intimate that they have induced the municipal authorities to prevent the appropriation of this "*surplus*" to the use of students of anatomy out of the city of New York. This ungenerous policy has already drawn down upon them the thunder of the press, and it is very unlikely that the New York Medical Colleges *out of that city* will quietly submit to such a course; and if it should bring difficulty, or even deprivation upon themselves, it may be to them a cause of future regret rather than exultation. A liberal and frank policy is always the best in the end.

We perceive by this Announcement that the number of the last Class was 407, instead of 425, as stated in the Buffalo Medical Journal and copied into our last number.

*Annual Announcement of the Medical Department of Pennsylvania College.*—Session 1846–7.

In the list of Medical Colleges published in our last number, the Medical Department of the Pennsylvania College was accidentally omitted. From this publication we learn that during the incumbency of the present Faculty, the Classes have been as follows, showing a rapid increase:

| | | | | |
|---|---|---|---|---|
| 1843–4 | Class | 23 | Graduates | 7 |
| 1844–5 | " | 60 | " | 14 |
| 1845–6 | " | 94 | " | 36 |

---

PHILADELPHIA COLLEGE OF PHARMACY.

We have been furnished by a friend with a pamphlet containing a *Report* of the Philadelphia College of Pharmacy, with a catalogue of its Members and Graduates.

The *Report* very justly says that "The length of time that has elapsed since the organization of the PHILADELPHIA COLLEGE OF PHARMACY, and its steadily increasing reputation and usefulness, have placed it among the established institutions in our country for the promotion of science." It is one of those institutions which have accomplished great good, without the least admixture of evil.

---

PRIVATE MEDICAL INSTITUTE.

We have received a *Circular* of a private Medical Institute, in Baltimore, conducted by our old friend Dr. J. R. W. Dunbar. We notice in this circular very ample means for the advancement of students, such as books, models, skeletons, preparations, &c., and are pleased to discover that a large number of gentlemen avail themselves of them. Dr. Dunbar is well qualified by temper and acquirements for the task he has assumed.

